# Metabolic and physiological effects of water stress on Moshgak (*Ducrosia anethifolia* Boiss) populations using GC–MS and multivariate analyses

**DOI:** 10.1038/s41598-022-25195-1

**Published:** 2022-12-22

**Authors:** Fatemeh Arabsalehi, Mehdi Rahimmalek, Mohammad R. Sabzalian, Amir Barzegar Sadeghabad, Adam Matkowski, Antoni Szumny

**Affiliations:** 1 Department of Agronomy and Plant Breeding, College of Agriculture, Isfahan University of Technology, Isfahan, 84156-83111 Iran; 2 Department of Horticulture, College of Agriculture, Isfahan University of Technology, Isfahan, 84156-83111 Iran; 3 Department of Horticulture, College of Agriculture, Shiraz University, Shiraz, Iran; 4 Department of Pharmaceutical Biology and Botany, Wroclaw Medical University, Borowska 211, 50-556 Wrocław , Poland; 5 Department of Food Chemistry and Biocatalysis, Wroclaw University of Life Sciences, Wrocław , Poland

**Keywords:** Physiology, Plant sciences

## Abstract

*Ducrosia anethifolia* Boiss is a medicinal and aromatic plant that is used as a food additive and drug in the areas of natural distribution. In this study, eight populations from different geographical regions of Iran were evaluated for their essential oil composition, total phenolics and flavonoids as well as for free radical scavenging activity during two consecutive years under water deficit. Analysis of variance was performed using a randomized block design at three levels of irrigation and 2 years, using the GLM procedure of SAS software and cluster analysis was used according to Ward's method using squared Euclidean dissimilarity. The highest essential oil and total phenolics/flavonoids content were obtained in the second year. GC–MS analysis was used to analyze the essential oil components. In normal conditions, *cis*-chrysanthenyl acetate (39.1–66.4%) and α-pinene (1.02–4.7%) were recognized as major compounds. The essential oil components were elevated under water stress. The highest increase in *cis*-chrysanthenyl acetate (21.32%) in response to water stress was observed in Abarkuh1. Elevation in total phenolic, flavonoid and antioxidant activity was also observed in plants subjected to water deficit. The highest content of phenolic acids was measured in Semirom (136.74 mg TAE/g DW), while the highest flavonoid content was in Kerman (6.05 mg QUE/g DW) in severe water stress condition. Finally, a high diversity in the studied populations can be used to select advantageous populations for other pharmaceutical and food purposes.

## Introduction

*Ducrosia* is a genus of flowering medicinal herbs belonging to the family *Umbelliferae*. *D. anethifolia* is one of the three *Ducrosia* species that is widely distributed in southern Egypt, Iran, Iraq, Afghanistan, and Pakistan^1^. This herb in Iran is generally known as Moshkbu and Moshgak^2^. The aerial parts of this aromatic medicinal plant have been applied as an analgesic reliever for backache, headache, colds, and colic^3^. Furthermore, Moshgak is added to many of foods for flavoring in some areas of Iran^2^.

Essential oils (EOs) are subtle, aromatic, and volatile liquids extracted from such plant materials as flowers, roots, bark, seeds, fruit peels, and wood^4^. Approximately 90% of global EO production is consumed by the flavor and fragrance industries, which is mostly in the form cosmetics, perfumes, soft drinks and food. In addition, more than 25% of the pharmaceutical drugs prescribed worldwide are derived from plant sources. Therefore, plant-originated products will be in increasing demand as researchers and producers continue to look for new plant sources^5^. Monoterpenes are the most common metabolites of essential oils in medical herbs^6^. However, Sefidkon and Javidtash^7^ identified decanal, α-thujene, decanol, sclareol, and limonene as the main components of dried aerial parts of Moshgak.

Today, the uses of many bioactive components such as phenolics and flavonoids is of the great value for human health^6^. Recently, many studies have reported the composition of essential oils, phenolics and flavonoids as well as their antioxidant activities in different medical herbs. The number of compounds and their bioactive capacities might be affected by genetic as well as environmental factors^8,9^. Drought is one of the main environmental factors that affects the concentration of metabolites^10^. Drought or osmotic stress leads to excessive generation of reactive oxygen species (ROS) that can be harmful to cellular components at high concentrations^11^. Plants demonstrate a wide range of stress tolerance or stress avoidance mechanisms to survive in a stress condition^10^. Plants under drought stress conditions mostly produce higher content of secondary metabolites compared with non-stressed condition^12^. There are many studies that highlight the effect of water stress on secondary metabolites of Apiaceae plants, including *Cuminum cyminum*^13^ and *Foeniculum vulgare*^14^. Total phenolic (TPC) and total flavonoids (TFC) and antioxidant activity can also be elevated in response to water stress in different medicinal plants^1,8,14,15^. Also, Mottaghipisheh et al.^16^ showed that the antioxidant activity of *D. anethifolia* in ethanolic and ethyl acetate extracts is less than Butylated hydroxytoluene (BHT) as a synthetic antioxidant that was used as positive control. Evaluation of populations in various years is one of the substantial segments of the breeding programs. Moreover, cultivation year can highly affect the secondary metabolites of medicinal plants such as *Foeniculum vulgare* Mill^9,17,18^ and *Stachys lavandulifolia*^19^.

There are limited studies on *D. anethifolia* populations. Sabbaghi and Rahimmalek^20^ assessed the morphological and molecular variation of Iranian Moshgak populations. Arbabi et al.^2^ also evaluated morphological and oil components of leaves in this species in one geographical region in Southeastern parts of Iran. However, no studies were performed on the effect of water stress on the composition of essential oils, phenolics, flavonoids, and antioxidant capacity of different populations of *D. anethifolia* in two consecutive years*.* As gas chromatography results provide valuable data with many information that is difficult to process based on univariate methods, multivariate analyses provide new tools for exploration and interpretation of chromatographic data^21–23^.

The purposes of this research were: (1) to evaluate different populations of *D.anethifolia* under experimental field conditions to select populations with high phytochemical contents; (2) to assess chemical composition and polyphenolic content in water stress condition and (3) to use multivariate analyzes for choosing and introduction of elite populations for further cultivation and processing.

## Results

### Effects of water stress and population on essential oil content

The ANOVA results showed significant differences between the Moshgak population studied for the essential oil content. Furthermore, the effect of year, irrigation, year × population interactions and irrigation × population interactions were significant for EOs (Table 1). The color of the extracted essential oil content was light yellow. However, the trends of essential oil over two consecutive years (Table 2) showed that the higher amount of the EOs occurred in the second year. The EOs content also increased under drought stress (Table 1).

**Table 1:** Analysis of variance (mean squares) for phytochemical traits of 24 Moshgak populations (P) evaluated at three levels of irrigation (I) and 2 years (Y) of 2019 and 2020.

SOV	DF	Total phenol	Total flavonoid	DPPH	Fruit essential oil
Y	1	1888**	63.79**	295,895**	28.64**
I	2	32,306**	49.28**	33,440**	11.08**
Y*I	2	33.23^ns^	0.109^ns^	1401^ns^	0.00001^ns^
R(Y*I)	12	27.77**	0.317**	1368^ns^	0.033^ns^
P	7	4627**	1.91**	749,727**	2.72*
Y*P	7	2.82^ns^	0.483**	4226*	0.257**
I*P	14	522.9**	1.92**	3263*	0.262**
Y*I*P	14	22.34*	1.14**	1261^ns^	0.021^ns^
Error	84	4.53	0.063	1294	0.019

DF: degrees of freedom; ns, non-significant; *, significant at P ≤ 0.05; **, significant at P ≤ 0.01.

**Table 2:** Mean comparisons of irrigation regimes, years and populations (interaction) on phytochemical traits of 8 Moshgak population.

Irrigation regimes	Population	Total phenolic (mg TAE/g DW)		Total flavonoid (mg QUE/ g DW)		DPPH (µg/ml)		Fruit essential oil (%)	
		2019	2020	2019	2020	2019	2020	2019	2020
Control	Abadeh	71.81	75.56	3.39	4.59	348.03	403.15	1.63	2.15
	Ferydunshahr	95.87	99.34	3.54	3.93	711.87	723.34	1.65	2.19
	Qazvin	59.21	64.29	3.07	3.65	349.31	375.19	1.56	2.06
	Kerman	45.18	47.62	4.13	4.68	187.57	236.15	2.27	2.74
	Semirom	90.28	98.90	3.48	3.85	674.44	730.13	1.31	1.85
	Hormozgan	72.22	78.37	3.80	4.58	166.17	216.63	1.46	1.93
	Abarkuh1	80.39	88.21	3.52	4.09	726.81	725.38	1.93	2.42
	Abarkuh2	45.49	51.00	3.75	4.26	351.50	422.17	2.42	2.85
Moderate water stress	Abadeh	74.68	77.37	3.59	5.28	338.79	393.91	1.93	2.41
	Ferydunshahr	99.79	102.3	4.09	4.53	700.78	715.00	1.84	2.49
	Qazvin	62.58	65.97	4.25	4.86	340.08	365.96	1.79	2.36
	Kerman	48.78	52.04	4.66	5.36	178.34	226.92	2.46	3.04
	Semirom	93.32	96.63	4.04	4.46	661.55	725.68	1.54	2.15
	Hormozgan	75.53	78.94	4.39	4.98	156.93	207.40	1.76	2.16
	Abarkuh1	84.32	87.21	4.02	4.52	718.05	729.65	2.23	2.65
	Abarkuh2	48.52	51.50	4.21	4.94	342.27	412.93	2.61	3.15
	Abadeh	111.17	115.4	4.81	5.97	315.53	370.65	2.46	2.94
Severe water stress	Ferydunshahr	103.50	108.71	4.60	5.13	678.13	692.35	2.17	2.82
	Qazvin	69.43	74.49	4.35	4.85	316.81	342.69	2.32	2.89
	Kerman	94.61	98.59	4.84	6.05	155.07	203.65	2.99	3.56
	Semirom	131.19	136.74	4.17	5.07	638.90	703.03	1.86	2.48
	Hormozgan	89.06	94.27	4.86	5.38	133.67	184.13	2.29	2.68
	Abarkuh1	120.25	125.90	4.95	5.16	695.40	707.00	2.56	2.98
	Abarkuh2	99.59	103.87	5.05	5.62	319.00	389.67	3.14	3.68
	LSD	3.46		0.40		58.44		0.22	

In each trait, means having difference less than the LSD provided are not significantly different (p < 0.05).

In the first agronomic year (2019), the highest and lowest increase in essential oil content was observed for populations of Abadeh (83%) and Ferydunshahr (52%), respectively (Table 2).

In the second agronomic year (2020), the water deficit caused significant increases in essential oil in all Moshgak populations. The highest EOs enhancement was observed in the Qazvin and Abarkuh2 populations (83%), while the lowest in Abarkuh1 (56%) (Table 2).

### Effects of drought stress and population on essential oil composition

The essential oil constituents are listed in Table 3. Chromatogram of GC–MS peaks corresponding to *D. anethifolia* and the mass spectra of major compounds are illustrated in Fig. S1. The results indicated a high chemical polymorphism among Moshgak populations, and the 26 EOs components were affected both by irrigation regime and population of origin. The main constituents detected in the EOs were *cis*-chrysanthenyl acetate and α-pinene, (Table 3). The results also showed that *cis*-chrysanthenyl acetate was the most abundant component. In control conditions, the highest and lowest amount of this compound was observed in Semirom (66.4%) and Abadeh (39.1%), respectively. Furthermore, the concentrations of the essential oil composition in all Moshgak populations elevated significantly under water stress (Table 3). The highest and lowest increases in *cis*-chrysanthenyl acetate were in Abarkuh1 (21.32%) and Hormozgan (6.42%), respectively (Table 3). Under water deficit, α-pinene the highest increase was in Kerman populations (6.1%) and the lowest in Qazvin (0.61%) (Table 3). The maximum increase in trans-verbenol was observed for the Abadeh population. Sabinene was not present in the Abadeh, Ferydunshahr1, and Abarkuh1 populations. However, in fruits from drought stressed plants the emergence of sabinene noticed (Table 3).

**Table 3:** The mean of essential oil composition of eight Moshgak populations evalutated at three levels of irrigation.

Component	RI*		Abadeh			Ferydunshahr1			Qazvin2			Kerman		
	Exp.	Lit.	C**	MS	SS	C	MS	SS	C	MS	SS	C	MS	SS
α-Thujene	929	952	nd***	nd	nd	nd	nd	nd	nd	nd	nd	nd	nd	nd
**α-Pinene**	**937**	**961**	**4.7**^h^****	**11.1**^**b**^	**12.6**^**a**^	**4.05**^**l**^	**4.4**^**j**^	**5.7**^**f**^	**3.28**^**o**^	**3.68**^**n**^	**3.89**^**m**^	**2.32**^**r**^	**7.11**^**d**^	**8.33**^**c**^
Thuja-2,4-(10)diene	956	980	0.23	0.38	1.08	nd	nd	0.62	nd	nd	0.54	nd	nd	nd
**Sabinene**	**974**	**1000**	**nd **	**0.61**^**fg**^	**0.84**^**b**^	**nd**	**0.33**^**m**^	**0.62**^**e**^	**0.47**^**j**^	**0.5**^**i**^	**0.70**^**c**^	**0.33**^**m**^	**0.60**^**g**^	**0.95**^**a**^
**β-Pinene**	**979**	**1009**	**0.42**^**i**^	**0.86**^**c**^	**1.02**^**b**^	**0.32**^**l**^	**0.40**^**j**^	**0.65**^**e**^	**0.33**^**kl**^	**0.49**^**h**^	**1.22**^**a**^	**nd**	**0.25**^**o**^	**0.60**^**f**^
**Myrcane**	**991**	**1012**	**1.27**^**g**^	**2.30**^**b**^	**2.55**^**a**^	**1.18**^**i**^	**1.23**^**h**^	**1.43**^**e**^	**nd**	**1.2**^**j**^	**1.32**^**f**^	**1.24**^**h**^	**2.15**^**c**^	**2.3**^**b**^
***P*-Cymene**	**1025**	**1054**	**0.35**^**m**^	**0.64**^**g**^	**0.99**^**e**^	**0.24**^**o**^	**0.25**^**o**^	**0.26**^**o**^	**nd**	**nd**	**1.06**^**c**^	**nd**	**nd**	**0.62**^**h**^
**Limonene**	**1030**	**1059**	**1.03**^**f**^	**1.51**^**c**^	**1.53**^**b**^	**0.74**^**l**^	**0.78**^**j**^	**0.87**^**h**^	**nd**	**1.18**^**e**^	**1.74**^**a**^	**0.58**^**o**^	**0.96**^**g**^	**1.21**^**d**^
**γ-Terpinene**	**1060**	**1086**	**0.22**^**n**^	**0.58**^**i**^	**1.31**^**d**^	**0.29**^**l**^	**0.31**^**k**^	**0.34**^**j**^	**nd**	**0.31**^**k**^	**2.9**^**a**^	**0.34**^**j**^	**0.78**^**f**^	**0.98**^**e**^
Linalool	1099	1121	0.20	0.38	0.46	nd	0.11	0.25	nd	nd	0.35	nd	nd	0.14
*Cis*-Verbenol	1142	1162	0.39	0.53	1.33	0.23	0.56	0.96	nd	nd	nd	0.3	0.38	1.93
***trans*-Verbenol**	**1144**	**1165**	**1.86**^**d**^	**2.80**^**b**^	**3.32**^**a**^	**0.42**^**s**^	**0.87**^**o**^	**1.39**^**i**^	**nd**	**0.93**^**n**^	**1.2**^**k**^	**0.55**^**r**^	**1.55**^**g**^	**1.93**^**c**^
*Cis*-Chrysantheol	1160	1175	nd	nd	1.95	nd	nd	0.47	nd	nd	0.49	nd	nd	0.35
*p*-Cymen-8-ol	1182	1216	nd	nd	0.22	nd	nd	nd	nd	nd	0.36	nd	nd	0.2
**Decanal**	**1205**	**1221**	**nd**	**nd**	**0.56**^**d**^	**nd**	**0.32**^**ij**^	**0.57**^**d**^	**nd**	**0.31**^**jk**^	**0.69**^**c**^	**nd**	**nd**	**0.35**^**h**^
Verbenone	1204	1233	nd	0.43	0.48	nd	0.15	0.37	nd	nd	nd	nd	0.32	0.38
***Cis*-Chrysanthenyl acetate**	**1264**	**1292**	**39.1**^**x**^	**43.8**^**v**^	**56.9**^**n**^	**54.3**^**p**^	**60.2**^**k**^	**67.65**^**d**^	**55.5**^**o**^	**61.41**^**j**^	**63.96**^**i**^	**53.6**^**q**^	**60**^**l**^	**64.3**^**h**^
*Trans*-Pinocarvyl acetate	1297	1324	nd	nd	2.08	nd	nd	1.47	nd	nd	nd	nd	nd	2.62
*Cis*-Pinocarvyl acetate	1312	1333	nd	nd	2.68	nd	nd	2.75	nd	nd	nd	nd	nd	nd
Myrtenyl acetate	1327	1347	nd	0.3	0.59	nd	nd	0.25	nd	nd	nd	nd	nd	nd
**Geranyl acetate**	**1382**	**1404**	**0.54**^**u**^	**1.07**^**h**^	**2.29**^**c**^	**0.54**^**t**^	**0.6**^**s**^	**0.65**^**p**^	**0.94**^**j**^	**1.24**^**g**^	**1.53**^**e**^	**0.52**^**v**^	**0.61**^**q**^	**0.75**^**n**^
*z*-Jasmonyl acetate	1452	1472	nd	0.3	0.73	0.27	0.3	0.37	0.22	0.24	0.28	nd	0.39	0.40
(2E) Dodecanal	1468	1485	nd	0.33	0.87	nd	0.43	0.58	0.45	0.52	0.94	nd	0.27	0.35
Monoterpene hydrocarbons	–	–	8.22	17.98	21.92	6.82	7.7	10.49	4.08	7.36	13.37	4.81	11.85	14.99
Oxygenated monoterpene	–	–	2.45	4.14	8.1	0.65	2.01	4.01	0	1.24	2.73	0.85	2.25	5.08
Others	–	–	39.64	45.8	66.36	55.11	61.53	73.72	57.11	63.41	67.07	54.12	61.27	68.62
Total amount of compounds	–	–	50.31	67.92	96.38	62.58	71.24	88.22	61.19	72.01	83.17	59.78	75.37	88.69

Component	RI*		Semirom1			Hormozgan3			Abarkuh1			Abarkuh2		
	Exp.	Lit.	C**	MS	SS	C	MS	SS	C	MS	SS	C	MS	SS
α-Thujene	929	952	nd***	nd	nd	nd	nd	nd	nd	nd	nd	nd	nd	0.24
**α-Pinene**	**937**	**961**	**2.41**^q^****	**3.15**^**p**^	**5.4**^**g**^	**3.9**^**m**^	**4.66**^**i**^	**6.7**^**e**^	**1.02w**	**1.44**^**u**^	**1.91**^**t**^	**1.12**^**v**^	**2.14**^**s**^	**4.07**^**k**^
Thuja-2,4-(10)diene	956	980	nd	nd	nd	nd	nd	nd	nd	nd	nd	nd	nd	0.45
**Sabinene**	**974**	**1000**	**0.53**^**h**^	**0.60**^**g**^	**0.70**^**c**^	**0.45**^**k**^	**0.52**^**h**^	**0.68**^**d**^	**nd**	**nd**	**0.35**^**l**^	**0.25**^**n**^	**0.6**^**g**^	**0.61**^**ef**^
**β-Pinene**	**979**	**1009**	**0.23**^**p**^	**0.34**^**k**^	**0.69**^**d**^	**0.27**^**n**^	**0.30**^**m**^	**0.41**^**ij**^	**nd**	**nd**	**nd**	**nd**	**nd**	**0.60**^**g**^
**Myrcane**	**991**	**1012**	0.47^n^	0.73^l^	1.46^d^	0.59^m^	0.74^k^	0.89^j^	nd	nd	0.21^o^	nd	nd	0.92^i^
***P*-Cymene**	**1025**	**1054**	0.58^i^	0.83^f^	0.84^f^	0.28^n^	0.38^k^	0.82^f^	0.23^p^	4.3^b^	4.97^a^	0.2^q^	0.43^j^	1.34^c^
**Limonene**	**1030**	**1059**	nd	0.35^q^	0.86^i^	0.68^m^	0.75^l^	1.04^f^	0.24^r^	0.63^n^	0.77^k^	nd	0.56^p^	0.68^m^
**γ-Terpinene**	**1060**	**1086**	nd	nd	0.77^fg^	nd	1.34^c^	1.4^b^	0.33^j^	0.68^h^	0.75^g^	nd	nd	0.27^m^
Linalool	1099	1121	nd	nd	0.35	nd	nd	nd	0.21	0.43	0.54	nd	0.28	0.38
*Cis*-Verbenol	1142	1162	nd	nd	0.20	0.29	1.11	1.26	0.55	0.72	0.82	nd	nd	0.82
***trans*-Verbenol**	**1144**	**1165**	**0.98**^**m**^	**1.25**^**j**^	**1.72**^**f**^	**1.18**^**l**^	**1.45**^**h**^	**1.81**^**e**^	**0.32**^**x**^	**0.38**^**v**^	**0.40**^**u**^	**0.34**^**w**^	**0.63**^**q**^	**0.75**^**p**^
*Cis*-Chrysantheol	1160	1175	nd	nd	nd	nd	nd	0.59	nd	nd	nd	nd	nd	0.24
*p*-Cymen-8-ol	1182	1216	nd	nd	nd	nd	nd	nd	nd	nd	nd	nd	nd	nd
**Decanal**	**1205**	**1221**	**0.34**^**hi**^	**0.43**^**g**^	**1.52**^**b**^	**0.33**^**hi**^	**0.46**^**f**^	**1.99**^**a**^	**0.23**^**l**^	**0.35**^**h**^	**0.5**^**e**^	**nd**	**nd**	**0.3**^**k**^
Verbenone	1204	1233	nd	nd	0.19	nd	0.17	0.38	nd	nd	0.17	nd	nd	nd
***Cis*-Chrysanthenyl acetate**	**1264**	**1292**	**66.4**^**f**^	**67.1**^**e**^	**71.1**^**a**^	**51.2**^**s**^	**51.9**^**r**^	**57.6**^**m**^	**43.4**^**w**^	**49.7**^**t**^	**64.7**^**g**^	**47.2**^**u**^	**67.9**^**c**^	**68.4**^**b**^
*Trans*-Pinocarvyl acetate	1297	1324	nd	nd	2.64	nd	nd	2.9	nd	nd	nd	nd	nd	2.37
*Cis*-Pinocarvyl acetate	1312	1333	nd	nd	0.64	nd	nd	2.68	nd	nd	nd	nd	nd	0.42
Myrtenyl acetate	1327	1347	nd	nd	nd	nd	nd	0.74	nd	nd	nd	nd	nd	nd
**Geranyl acetate**	**1382**	**1404**	**0.42**^**x**^	**0.61**^**r**^	**1.42**^**f**^	**0.49**^**w**^	**0.67**^**o**^	**0.86**^**k**^	**0.83**^**l**^	**1.03**^**i**^	**3.03**^**a**^	**0.82**^**m**^	**2.15**^**d**^	**2.36**^**b**^
*z*-Jasmonyl acetate	1452	1472	nd	nd	0.49	nd	nd	0.21	0.25	0.35	0.59	nd	0.21	0.24
(2E) Dodecanal	1468	1485	nd	0.37	0.49	nd	0.36	0.53	0.34	0.51	0.54	nd	nd	0.44
Monoterpene hydrocarbons	–	–	4.22	6	10.72	6.17	8.69	11.94	1.82	7.05	8.96	1.57	3.73	11.27
Oxygenated monoterpene	–	–	1.32	1.68	3.98	1.8	3.19	6.03	1.31	1.88	2.43	0.34	0.91	2.49
Others	–	–	66.82	68.08	76.78	51.69	52.93	65.52	44.82	51.59	68.86	48.02	70.26	74.23
Total amount of compounds	–	–	72.36	75.76	91.48	59.66	64.81	83.49	47.95	60.52	80.25	49.93	74.9	87.99

Significant values are in bold.

*The data were sorted based on the retention index (RI) of components. **C: Control; MS: Moderate Stress; SS: Severe Stress. ***nd: Not detected. Literature indexes were obtained from NIST database (2009). ****The means were significantly different at 5% probability level.

Mean with the different superscript letters in a column are different significantly (p < 0.05).

### Effects of drought stress and population on phenolic contents

The results of the analysis of variance revealed a significant variation between the Moshgak population studied for the total phenolic content (TPC) and flavonoid content (TFC). In the present study, TPC and TFC were affected by year and irrigation (Table 1). The interaction effects were significant between the population × irrigation and the year × irrigation × population for these characteristics.

During the first season of experiment, under control conditions, “Ferydunshahr” exhibited the highest TPC (95.87 mg of TAE/g of DW), while 'Kerman' showed the highest TFC (4.13 mg of QE/g of DW). Elevated total phenolic and flavonoid content in all populations under drought stress (Table 2).

In the second year, under control conditions, the highest TPC was observed in Ferydunshahr (99.34 mg of TAE/g of DW), whereas the maximum TFC was recorded in Kerman (4.68 mg of QE/g of DW). Drought stressed samples from all populations had significantly increased total phenolic and flavonoid content. The highest increase of TPC and TFC were in populations of Abarkuh (52.87%) and Kerman (1.37%), respectively (Table 2).

### Effects of drought stress and population on DPPH radical scavenging assay

The results of ANOVA indicated a significant difference among the Moshgak population studied for the DPPH radical scavenging assay (IC_50_). Furthermore, the effect of year, irrigation, population year × population irrigation × population interactions was significant for IC_50_ (Table 1). In the first year, under control conditions, the highest antioxidant activity (the lowest IC_50_ value) was obtained in Hormozgan (166.17 µg/ml), while Abarkuh1 possessed the lowest antioxidant activity (the lowest IC_50_ value) (726.81 µg/ml). Under drought stress antioxidant activity increased (decreased IC_50_) in all Moshgak populations (Table 2).

In the second year, under control conditions, the lowest and highest IC_50_ were observed in Hormozgan (216.63 µg/ml) and Semirom (730.13 µg/ml), respectively (Table 3). Additionally, the activity increased in response to drought stress (Table 2).

### Correlations between the main essential oil components and TPC and TFC

The correlation coefficients among the main components of essential oil and total content of phenolics and flavonoids are presented in Table 6. In control conditions, the highest coefficeints were between α-pinene and β-pinene (r = 90**), as well as myrcane and limonene (r = 84**). *Cis*-chrysanthenyl acetate had a weak positive correlation with β-pinene (r = 0.66*), but was slightly negatively correlated with limonene (r = − 0.46*) (Table 6). Furthermore, the relationship between p-cymene and total phenolics (r = 66**), and IC_50_ (r = 0.48 *) was positive, but the correlation was negative with essential oil content (r = − 0.54*). However, there was no significant correlation between *cis*-chrysanthenyl acetate and flavonoids or phenolics content (Table 4).

**Table 4:** Pearson correlation coefficients between major essential oil compounds of fruit essential oil, total phenol, total flavnoid and DPPH of eight Moshgak population in control (lower triangle) and moderate water stress (upper triangle) conditions.

Compounds	1	2	3	4	5	6	7	8	9	10	11	12	13	14	15
α-Pinene	1	0.49*	0.81**	0.9**	− 0.4	0.79**	0.29	0.95**	− 0.51*	− 0.55*	− 0.26	− 0.08	− 0.12	0.31	− 0.44
Sabinene	0.03	1	0.4	0.46*	− 0.83**	0.2	− 0.15	0.58*	− 0.41	0.31	0.1	− 0.01	-0.43	0.47*	− 0.59*
β-Pinene	0.9**	0.09	1	0.78**	− 0.44	0.77**	0.05	0.83**	− 0.13	− 0.43	− 0.3	− 0.53*	0.2	− 0.07	− 0.17
Myrcane	0.66**	− 0.39	0.35	1	− 0.51*	0.76**	0.21	0.8**	− 0.43	− 0.33	− 0.43	− 0.1	− 0.12	0.41	− 0.40
*p*-Cymene	0.12	− 0.13	0.29	0.07	1	− 0.28	0.1	− 0.39	0.26	− 0.39	0.02	0.08	0.31	− 0.49*	0.55*
Limonene	0.69**	− 0.56*	0.43	0.84**	0.03	1	0.25	0.68**	− 0.46*	− 0.59*	0.008	− 0.04	− 0.25	0.26	− 0.45*
γ-Terpinene	− 0.05	− 0.72**	− 0.24	0.52*	− 0.26	0.51*	1	0.28	0.18	− 0.66**	− 0.43	− 0.12	− 0.05	0.45*	− 0.53*
trans-Verbenol	0.57*	− 0.25	0.48*	0.55*	0.6**	0.66**	− 0.02	1	− 0.38	− 0.53*	− 0.26	− 0.25	0.05	0.22	− 0.43
Decanal	− 0.16	0.24	− 0.05	− 0.28	0.62**	− 0.19	− 0.31	0.23	1	0.03	− 0.48*	− 0.74**	0.69**	− 0.47*	0.40
*Cis*-Chrysanthenyl acetate	0.016	0.66**	0.06	− 0.06	0.23	− 0.46*	− 0.38	− 0.27	0.34	1	0.24	0.18	− 0.18	0.06	0.13
Geranyl acetate	− 0.47*	0.09	− 0.26	− 0.7**	− 0.57*	− 0.53*	− 0.11	− 0.67**	− 0.35	− 0.33	1	0.56*	− 0.57*	0.002	− 0.13
Seed essential oil	− 0.60**	− 0.31	− 0.8**	− 0.07	− 0.54*	− 0.12	0.35	− 0.38	− 0.53*	− 0.4	0.36	1	− 0.7**	0.47*	− 0.23
Total phenol	0.25	− 0.26	0.44	0.12	0.68**	0.15	0.10	0.21	− 0.50*	0.25	− 0.37	− 0.71**	1	− 0.79**	0.75**
Total flavonoid	− 0.02	− 0.27	− 0.41	0.48*	− 0.12	0.50*	0.32	0.38	0.0007	− 0.31	− 0.46**	0.48*	− 0.43	1	− 0.87**
DPPH	− 0.27	− 0.38	− 0.08	− 0.08	0.48*	− 0.25	0.24	− 0.21	0.22	0.2	0.03	− 0.22	0.76**	− 0.56*	1

*Significant at P ≤ 0.05; **significant at P ≤ 0.01.

In moderate water stress conditions, the Pearson correlation matrix indicated a significantly positive correlation between α-pinene and *trans*-verbenol (r = 0.95**), myrcane (r = 0.90**), β-pinene (r = 0.81**) and limonene (r = 0.79**) (Table 5). Furthermore, the correlation between *cis*-chrysanthenyl acetate and α-pinene (r = − 0.55*), limonene (r = − 0.59*), γ-terpinene (r = − 66**), and trans-verbenol (r = − 0.53*) were negative (Table 4).

Decanal was negatively correlated with essential oil (r = − 0.74**) and TFC (r = − 0.47*), but it was positively correlated with TPC (r = 0.69**). Furthermore, no significant correlation was found between *cis*-chrysanthenyl acetate and phytochemical traits (Table 4).

In severe water stress conditions, α-pinene had a significantly positive correlation with trans-verbenol (r = 0.97**), myrcene (r = 0.87**) and sabinene (r = 0.83**), but the correlation was negative with *cis*-chrysanthenyl acetate (r = − 0.58*) (Table 6). Also, *cis*-chrysanthenyl acetate had a negative correlation with *trans*-verbenol (r = − 0.57*) and limonene (r = − 0.54*), but it was slightly positively correlated with IC_50_ (r = 0.61**) (Table 5).

**Table 5:** Pearson correlation coefficients between major essential oil compounds of fruit essential oil, total phenol, total flavonoid and DPPH of eight Moshgak population severe water stress conditions.

Compounds	1	2	3	4	5	6	7	8	9	10	11	12	13	14	15
α-Pinene	1														
Sabinene	0.83**	1													
β-Pinene	0.45*	0.33	1												
Myrcane	0.87**	0.86**	0.65**	1											
*p*-Cymene	− 0.53*	− 0.68**	− 0.65**	− 0.64**	1										
Limonene	0.46*	0.24	0.79**	0.57*	− 0.31	1									
Ɣ-Terpinene	0.04	− 0.12	0.61**	0.13	− 0.1	0.86**	1								
trans-Verbenol	0.97**	0.77**	0.53*	0.85**	− 0.54*	0.54*	0.17	1							
Decanal	0.02	0.019	− 0.09	− 0.2	− 0.21	− 0.06	0.17	0.17	1						
Cis-Chrysanthenyl acetate	− 0.58*	− 0.24	− 0.13	− 0.26	0.01	− 0.54*	− 0.42	− 0.57*	− 0.19	1					
Geranyl acetate	− 0.24	− 0.50*	− 0.24	− 0.36	0.77**	− 0.14	− 0.08	− 0.26	− 0.37	− 0.02	1				
Seed essential oil	0.004	0.2	− 0.14	0.08	0.1	− 0.11	− 0.24	− 0.2	− 0.68**	0.006	0.23	1			
Total phenol	0.02	− 0.006	− 0.45*	− 0.06	0.34	− 0.58*	− 0.66**	0.05	0.07	0.34	0.35	− 0.27	1		
Total flavonoid	0.50*	0.52*	− 0.24	0.32	0.03	− 0.09	− 0.37	0.3	− 0.38	− 0.44	0.17	0.74**	− 0.009	1	
DPPH	− 0.44	− 0.52*	− 0.28	− 0.36	0.4	− 0.47*	− 0.42	− 0.37	− 0.1	0.61**	0.28	− 0.44	0.68**	− 0.52*	1

*Significant at P ≤ 0.05; **significant at P ≤ 0.01.

### Hierarchical cluster analysis and principal component analysis

**Figure 1:**
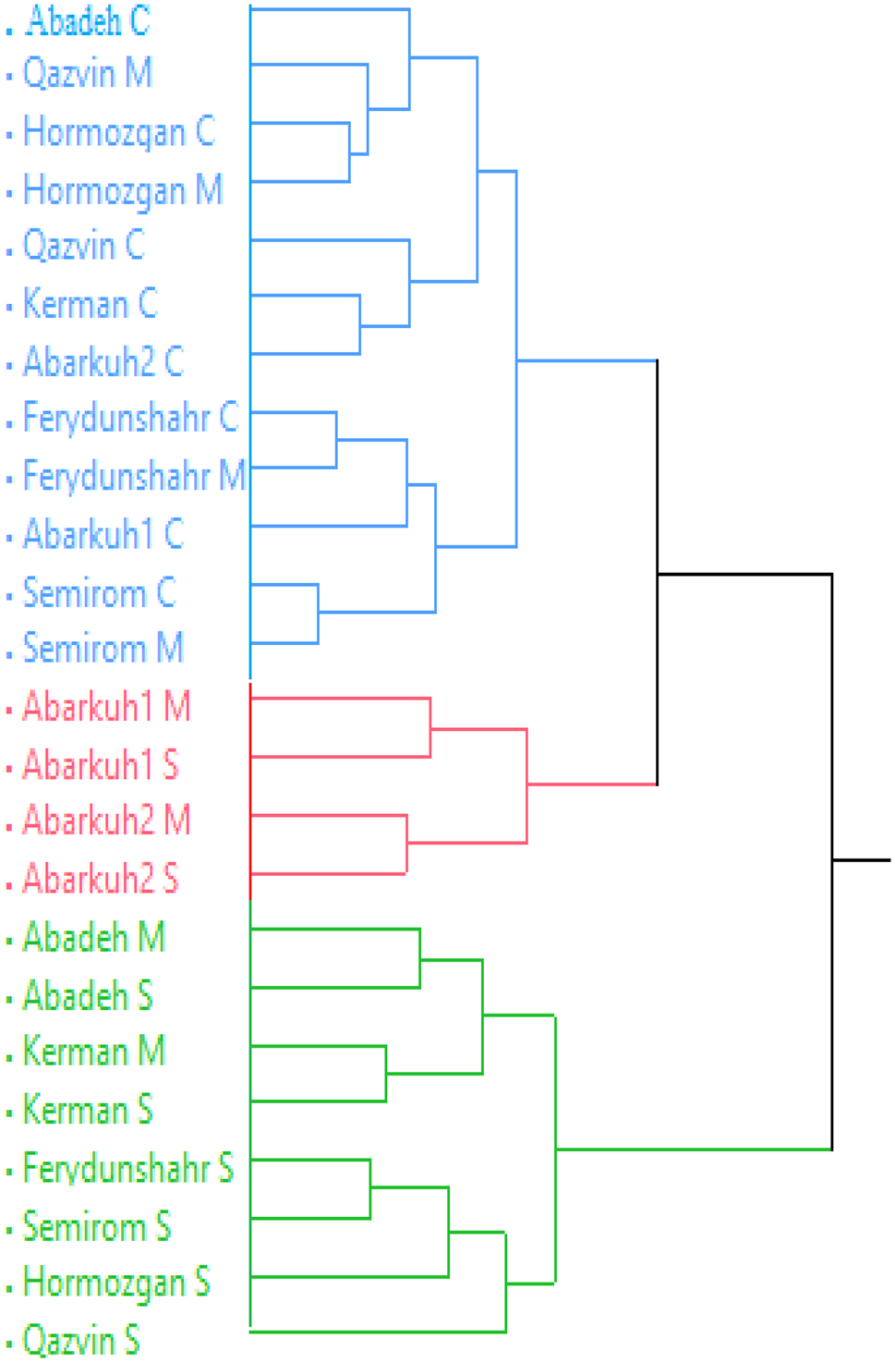
Dendrogram generated from cluster analysis of 8 Moshgak populations in three irrigation regimes based on phytochemical characters using WARD based on the Squared Euclidean dissimilarity calculated.

To investigate the likely similarities and relationships between the populations, a hierarchical cluster analysis (HCA) was performed based on the average of 2 years (Fig. 1). The HCA results can be applied to choose the superior parents based on the suitable genetic distance and *cis*-chrysanthenyl acetate for targeted crossing in future Moshgak breeding programs. According to the HCA, the Moshgak populations studied were grouped into three groups. Cluster I comprised 12 populations of normal and moderate water stress conditions. The second group included Abarkuh populations from moderate and severe water stress conditions with the highest *cis*-chrysanthenyl acetate, p-cymene, geranyl acetate, total phenol and IC_50_. Eight populations from moderate and severe water stress conditions were placed in the third group. They were distinguished by high α-pinene, sabinene, β-pinene, myrcane, limonene, γ-terpinene, trans-verbenol, decanal, and total flavonoids (Fig. 1).

**Figure 2:**
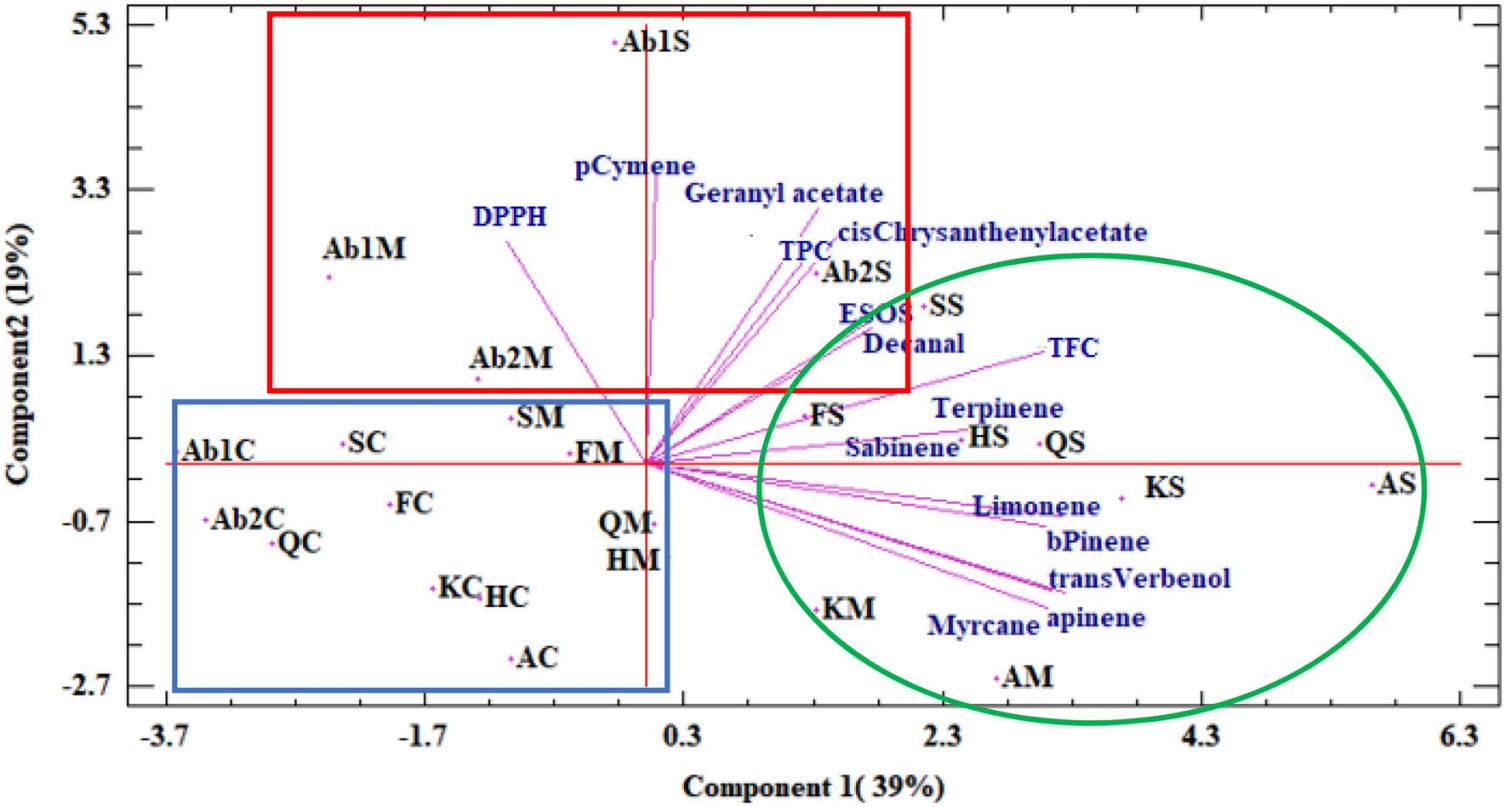
PCA analysis based on phytochemical characters in eight Moshgak populations in three Irrigation regimes.

The principal component analysis (PCA) was performed based on the main phytochemical traits averaged for 2 years. The principal component analysis confirmed the results of HCA (Fig. 2). The PCA result indicated that the first two components explained 39% and 19% of the total variation, respectively (Fig. 2). The first PC (PC1) had a positive association with α-pinene, sabinene, β-pinene, myrcane, limonene, γ-terpinene, *trans*-verbenol, decanal and flavonoids. The second PC (PC2) had a high correlation with the essential oil content, *cis*-chrysanthenyl acetate, p-cymene, geranyl acetate and total phenolic and IC_50_. The Abarkuh1 and Abarkuh2 populations in moderate and severe water stress conditions were classified in the same group. Abadeh and Kerman populations under moderate and severe water stress conditions were classified near each other. Additionally, Ferydunshahr, Semirom, Hormozgan,, and Qazvin populations under severe water stress conditions were classified in the same group. The others are classified into a separate group. Based on the biplot analysis, selection according to the high PC1 and PC2 values leads to populations with high *cis*-chrysanthenyl acetate, geranyl acetate, and total phenolics (Fig. 2).

## Discussion

Medicinal herbs have been used for thousands of years by many people all over the world. Due to the overharvesting of natural habitats, the expansion of procedures for preservation, assessment and application of medicinal herbs is essential for the protection and utilization of genetic resources^18^. Narrow information on genetic diversity and the effect of drought stress on Moshgak is available. In this study, we analyze various Moshgak populations collected from different geographical regions of Iran by phytochemical traits and essential oil components under drought stress conditions. The results of this study presented significant differences among populations and irrigation regimes for all characteristics studied, representing a wide diversity among populations in response to drought stress in this germplasm. Previously, a wide variation in important morphological traits and oil content of local Moshgak populations was reported^2,7^. The results of this research showed that populations are significantly different for EOs and reacted differently in response to drought stress. The increase in EOs production in response to a water deficit may be due to carbon reabsorption, as plant growth is reduced, for the biosynthesis of stress-related metabolites^24^. Previous studies reported different ranges of essential oil content in leaves of Moshgak populations^16,25^, while in the present study, the oil content ranged from 1.31 to 3.68% in fruits. EOs content can be influenced by environmental factors^4,19^. Alinian and Razmjoo^26^ observed that drought stress can lead to an increase in Cumin EO (*Cuminum cyminum* L.). In addition, Bettaieb et al.^13^ reported that fruits essential oil decreased in cumin in response to severe drought stress, while it increased in response to moderate conditions of water deficit. These findings indicated that the EOs depend on genotypes, and drought level. The results of the GC–MS analysis indicated a wide chemical polymorphism among the Moshgak populations. In the present study, the oil components fruits of this plant were determined for the first time. Arbabi et al.^2^ reported that *cis*-chrysanthyl acetate, n-decanal, decanol, and α-pinene were the main compounds of leaves in populations of *D. anethifolia.* Furthermore, Karami and Bahloli^27^ reported n-decanal, dodecanal, *cis*-chrysanthenyl acetate, and α-pinene as the main compounds in the essential oil of *D. anethifolia* leaves. In the present study, *cis*-chrysanthyl acetate and α-pinene were the main compounds. It seems that the production of *cis*-chrysanthenyl acetate starts only after flower appearance. As the production of *cis*-chrysanthenyl acetate in the leaves was very low, the biosynthesis of this compound was carried out mainly in the full grown inflorescence^28^. Prostaglandin synthetase is inhibited by chrysanthenyl acetate and could have analgesic properties^29^. Furthermore, chrysanthenyl acetate might contribute to antimigraine activity, due to its inhibition of prostaglandin^30^. *Cis*-chrysanthyl acetate is a monoterpene hydrocarbon^2^. Previous studies revealed that monoterpenes can be highly influenced by environmental stress^31^. Plants produce high concentrations of monoterpenes under environmental stress conditions because of a low allocation of carbon to growth, suggesting a competition between defense and growth^32^.

In the present study, some of essential oil compounds increased in response to drought stress. In line with our findings, Bettaieb et al.^13^ reported that the application of irrigation regimes can cause significant changes in the cumin EOs quality. For example, the percentage of γ-terpinene increased significantly in response to drought stress. The variation in EO compounds at various levels of water deficits is due to changes in the activity of linked biosynthesis enzymes in response to water deficit^13^. Monoterpene formation is catalyzed by terpene synthases, which are activated by developmental and stress-linked factors^33^.

In this study, the total phenolic and total flavonoid content increased in response to water shortages, though they pursued various trends, suggesting that the biochemical responses of Moshgak to drought stress could be population dependent. Similarly, the phenolic contents of cumin under water stress increased greatly^13^. In contrast, Neffati et al.^34^ reported that the polyphenol content decreased under salinity stress in coriander (*C. sativum* L). The different trends in phenolic fractions under drought stress can be imputed to the synthesis of various components of TPC and TFC as efforts by various populations to adapt to different soil moisture levels. Many researchers reported that the accumulated components of TPC and TFC could be increased under drought stress as a means of improving crop quality in medicinal plants^7^. Harrison and Were^35^ reported that improved TPC and TFC in response to drought stress could be the result of the degradation of bigger phenolic components into smaller ones. Furthermore, phenolics have strong antioxidant activity, and therefore participate in advocacy versus reactive oxygen species (ROS) that are inevitably produced when photosynthetic metabolism is devastated by environmental constraints^13^. The results of the present study indicated that the populations are conspicuously different for IC_50_ and reacted differently in response to drought stress. Furthermore, variation was observed in the antioxidant activity of drought-stressed plants according to those of their TPC. In corroborating this finding, Bettaieb et al.^36^ indicated that the variation in the antioxidant activity of cumin was reflective of the variation in its TPC. Other researchers reported that cumin antioxidant activities exhibited that treated seeds with moderate and severe water deficit showed the maximum antioxidant activity of cumin^13^. Exposure of plants to adverse environmental conditions like salinity, extreme drought, or temperature stresses can enhance the production of ROS^13^. Low molecular weights antioxidants can effectively scavenge harmful radicals^11^.

In the present study, positive relationships were also observed between IC_50_ and TPC under drought conditions. Compared to our finding, previous studies indicated a significant correlation between antioxidant activity and TPC in *C. cyminum*^13^ and *C. sativum*^34^.

The results of the present study led to identified populations with high *cis*-chrysanthenyl acetate, essential oil from seeds, and total phenolic content using PCA (high PC1 and PC2). The Abarkuh populations under stress were classified into a separate group due to the high p-cymene content. Hosseini et al.^18^ reported that selection based on a biplot led to the identification of genotypes with high essential oil yield and main components in fennel.

Various relationships were obtained among the characteristics. Positive correlation of some compounds showed that selection according to one of the compounds can cause improvement of another one^18^. The results of the present study revealed that α-pinene had a negative correlation with *cis*-chrysanthenyl acetate. In addition, a negative correlation was observed between *cis*-chrysanthenyl acetate with *trans*-verbenol and limonene. Negative associations between some isomer compounds were also reported in previous research^4,6^.

The water deficit induces partial stomatal closure and massively reduces the CO_2_ influx into the leaves, which directly leads to a passive increase of all processes consuming NADPH^+^H^+^, including the biosynthesis of highly reduced secondary plant products. Alternatively, enzymes responsible for the biosynthesis of natural products could be actively up-regulated. The corresponding increase in NADPH^+^H^+^ consumption significantly leads to the dissipation of the stress-related energy and thus prevents the generation of oxygen radicals. These coherences reveal the stress mechanism that leads to increased biosynthesis of natural products such as isoprenoids, alkaloids, or phenolics^37^.

## Conclusion

The Moshgak germplasm used in the current study indicated a wide range of variations in phytochemical traits and essential oil compounds that can be useful for further breeding programs. The total phenolic and total flavonoid content, antioxidant activity and EOs increased in response to water shortages. Also, the essential oil compounds increased in response to drought stress. Furthermore, *cis*-chrysanthenyl acetate was the most abundant essential oil component in the fruits of this plant. The highest increase in *cis*-chrysanthenyl acetate (21.32%) in response to water stress was observed in Abarkuh1.Significant differences in response to drought stress revealed that each population can be exploited for certain purposes.

Superior populations introduced in the current research are suitable candidates for the further development of new cultivars for medicinal and pharmaceutical programs.

## Materials and methods

Ethics statement. This study was carried out in accordance with the Ethics Committee of Isfahan University of Technology, Iran. Written, informed consent was obtained from all volunteers. All experimental protocols were approved by the Ethical Committee in Isfahan University of Technology, Iran.

### Plant material and experimental condition

**Figure 3:**
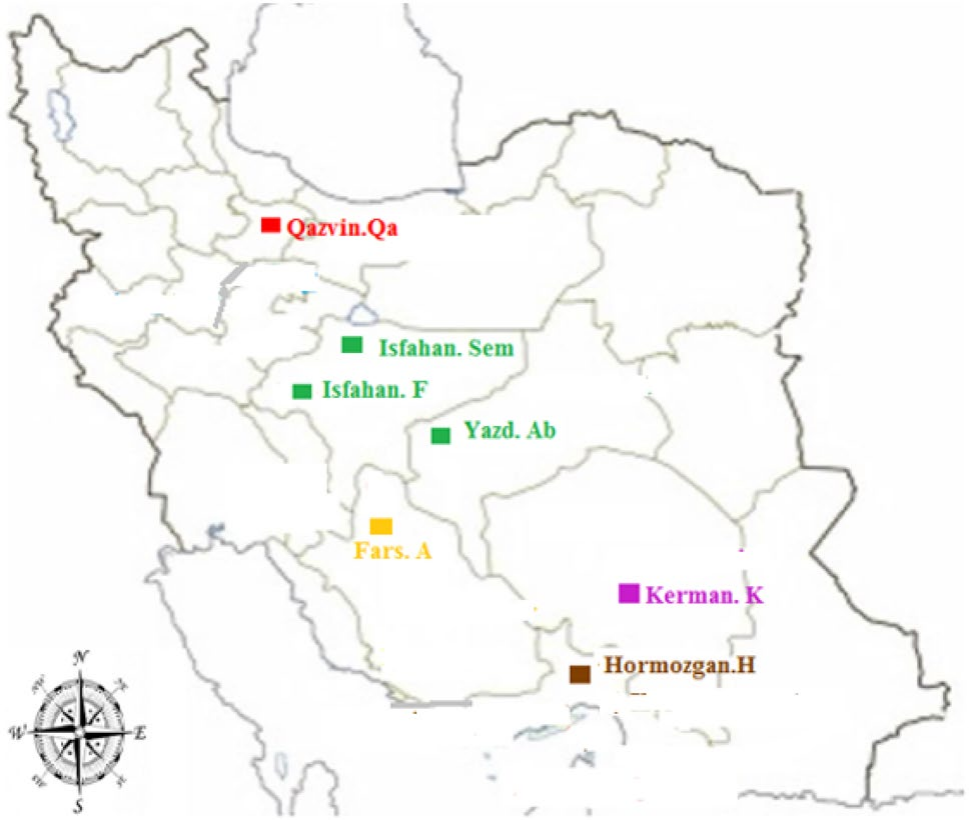
Sites of collection of Moshgak population from different geographical regions of Iran, used in this study. The population gene bank number are shown in Table 1.

The seeds of eight populations were obtained through medicinal gene bank of Core Research Facilities (CRF), Isfahan University of Medical Science, Isfahan, Iran. The collection of the samples were permitted from research institute of forest and rangelands and complies with local and national guidelines and legislation. The determination of plants was performed by Dr. Mehdi Rahimmalek and the samples were deposited in the herbarium of Isfahan university of technology, Isfahan, Iran. The geographical location, gene bank number and their characteristics of populations are shown in Table 6 and Fig. 3. The experiment was carried out on the IUT Research Farm, Esfahan (Latitude of 32° 38ʹ North, Longitude of 51° 39ʹ East). Seeds of all populations were planted in a Randomized Complete Block Design (RCBD with three replicates and three irrigation regimes (three treatments). Each plot included two rows of 2 m in length and 70 cm apart. The soil was clay loam texture, pH 7.5 and organic matter content of 1%. Fertilizers were used at 100 kg N/ha and 100 kg P/ha prior to sowing. Irrigation regimes were specified according to the procedure of Allen et al.^37^. The experimental research and field studies were performed based on the standard methods as described by the previous literatures^18,32,36^.

**Table 6:** Geographical location and their characteristics of 8 Moshgak populations.

Population code	Location	Herbarium number	Geographical region	latitude	longitude	Altitude(m)
A	Abadeh, Fars, Iran	DA-300	Southwest	31° 44′N	52° 31′E	203
H	Hormozgan, Hormozgan, Iran	DA-315	South	27° 48′N	57° 16′E	9
F	Fereydunshahr, Isfahan, Iran	DA-291	Center	33° 94′N	50° 12′E	2530
Sem	Semirom, Isfahan, Iran	DA-318	Center	31° 25′ N	51° 34′E	2398
Ab	Abarkuh, Yazd, Iran	DA-314	Center	31° 12′N	53° 28′E	1550
Ab	Abarkuh, Yazd, Iran	DA-316	Center	31° 12′N	53° 28′E	1550
Qe	Qazvin, Qazvin, Iran	DA-297	North	36° 26′N	50° 16′E	1347
K	Kerman, Kerman, Iran	DA-305	Southeast	30° 16′N	57° 04′E	2009

### Water stress treatment

Before irrigation, soil samples were obtained at soil depth of 0–15, 15–30, and 30–45 cm from three environments to estimate the soil water content and to compute the irrigation water content based on a 45-cm rooting depth. The irrigation depth was estimated according to the following formula:

$$ {\text{I}}\, = \,\left[ {\left( {{\text{FC}} - \uptheta } \right)/100} \right]{\text{ D}}\, \times \,{\text{B}}); $$

where, I is the depth of irrigation (cm), FC (− 0.03 MPa) is soil gravimetric moisture at field capacity (22%), θ (− 1.5 MPa) is soil gravimetric moisture percentage at irrigation time (10%), D is root-zone depth (60 cm), and B is soil bulk density of the soil in the root zone (1.4 g cm^−3^)^38^. Seeds were sown under three experimental conditions, including control condition (C), moderate (MS), and severe water stress (SS). The plants were irrigated at the sowing time, after which irrigation was carried out every week until the early flowering stage (20% flowering). Moreover, irrigation was continued until 40, 60 and 80%, respectively, of the field capacity (FC). To obtain the volume of each water application, a Parshall flume formula (Id = I × p; where p is the fraction of I that may be depleted from the root zone) was applied. Finally, Ig = (Id/Ea) × 100, with Ea representing irrigation efficiency (%), was supposed to be 65% on average.

### Essential oil extraction

Ripe fruits from *D. anethifolia* plants grown in each agronomic year were collected and dried in the shade at 25 °C for 4 days. The fruits of ten samples from each population were mixed and then applied to measure essential oils. The oil was obtained by hydro-distillation using a Clevenger-type apparatus for 4 h. For this purpose, 50 g of each samples was applied. Distilled essential oil was procured using diethyl ether as collecting solvent (v/v), collected in the glass container, and kept at 4 °C until analysis. Finally, the essential oil content was presented as percentage based on the weight of the air-dried seed specimens.

### Gas chromatography–mass spectrometry (GC–MS) analysis

Essential oil compositions were analyzed using an Agilent 7890 gas chromatograph (Agilent Technologies, Palo Alto, CA, USA) with a HP-5 MS capillary column (30 m × 0.25 mm, 0.25 μm film thickness). The oven temperature was programmed at 70 °C for 2 min followed by an increase to 200 °C (Hold = 1 min). Injector and detector temperatures were set at 210 and 220 °C, respectively. The analyzes were done via helium as the carrier gas (inlet pressure 17.7 psi; split 1:10). The gas chromatograph was coupled to the Agilent 5975C mass spectrometer as a selective detector with an ionization voltage of 70 eV; ion source and mass quadrupole temperatures of 230, and 150 °C, and mass range of 35–500 m/z. The GC MSD Chem Station program was applied as operating software. Retention indices were obtained by using retention times of n-alkanes (C8-C21) injected before essential oil analysis in the same conditions, using the Kovats index formula^39^. Essential oil constituents were identified by comparison of retention indices (RIs) with those reported in the literature^39^, as well as comparing their mass spectra with those recorded in the NIST 08 (National Institute of Standards and Technology), Wiley275. L (ChemStation data system). The percentage of composition was obtained on the peak areas of the total oil compositions.

### Methanolic extract and total phenolic content

The total phenolic content was obtained with the Folin–Ciocalteu reagent as explained in Hodaei et al.^8^ with some changes. Briefly, to provide a sample extract, 25 mL of 80% methanol was added and shaken at 25 °C for 24 h. The extract was filtered, and 2.5 mL of the Folin-Ciocalteu reagent (1:10 diluted with distilled water) and 2 mL of 7.5% sodium carbonate was added to 0.5 mL of the methanolic extract and shaken well. The mixture was heated at 45 °C for 15 min. Finally, the absorbance of the extract was read at 765 nm via spectrophotometer. The water and reagents was applied as a blank sample. Tannic acid was applied as the reference standard and the total phenolic content was reported as mg of tannic acid equivalents (TAE) equivalent per gram of sample on a dry basis.

### Total flavonoid content of the extracts

The aluminum chloride colorimetric procedure was used for the determination of total flavonoids by Sarfaraz et al.^40^ with minor changes. The methanolic extract (125 µL) was mixed with 75 µL of a 5% NaNO_2_ solution and after six minutes of keping in the dark, 150 µL of AlCl_3_ (10%) was added and incubated for 5 min. Then, 750 μL of NaOH (1 M) and 2500 μL of distilled water were added. After 15 min of incubation, the extract turned pink and the absorbance was read at 510 nm. Finally, TFC was reported in mg of quercetin equivalents (QE) per gram of fruits dry weight.

### DPPH radical scavenging assay

The radical scavenging activity of *D. anethifolia* was determined using the procedure of by Sarfaraz et al.^40^ with minor changes. Briefly, 5 mL of 0.1 mM methanol DPPH solution with 0.1 mL of the extract was mixed at selected concentrations (50, 100, and 300 ppm). The mixture was shaken and kept for 30 min at 25 °C. The absorbance of the samples was read using a UV–visible spectrophotometer at 517 nm with methanol (80%) as a blank and butylated hdroxytoluene (BHT) applied as standard antioxidant at 1–100 μg/ml for comparison. The absorbance was measured at 517 nm versus the blank. The percentage of inhibition of the samples was estimated on the basis of the following formula:

$$\mathrm{\%inhibition }=\frac{{(A}_{B}-{A}_{A})}{{A}_{B}}\times 100$$

where, ${A}_{A}$ and ${A}_{B}$ are the absorbance values of the DPPH radical in percentage in the fruits extract sample and the control, respectively. Subsequently, the inhibition percentage was plotted against the extract concentration and 50% of the inhibitory concentration (IC_50_) of the DPPH values was calculated (Fig. S1).

### Statistical analysis

The normality of the numerical data was computed using the proc normality test of SAS software release 9.2 (SAS Institute, Cary, NC, USA)^41^. Analysis of variance (ANOVA) was performed using a randomized block design at three levels of irrigation and 2 years, using the GLM procedure of SAS software release 9.2 (SAS Institute, Cary, NC, USA)^37^. Mean comparisons were made using the least significant difference (LSD) test (p < 0.05) using SAS software release 9.2 (SAS Institute, Cary, NC, USA)^37^.

Cluster analysis was used according to Ward's method using squared Euclidean dissimilarity through the Stat Graphics software ver. 17.2 (http://www.statgraphics.com). Principal component analysis (PCA) was performed to recognize the association among *D. anethifolia* populations and all measured characteristics using Stat Graphics software ver. 17.2 (http://www.statgraphics.com).

## Supplementary Information

Supplementary Information.

Supplementary Figure S1.

## Data Availability

The datasets used and/or analyzed during the current study are available from the corresponding author based on reasonable request.
