# Supplementary material for: Metabolic and physiological effects of water stress on Moshgak (Ducrosia anethifolia Boiss) populations using GC–MS and multivariate analyses
Source: Sci Rep. 2022 Dec 22;12:22148. doi: 10.1038/s41598-022-25195-1 (PMC9780208; doi:10.1038/s41598-022-25195-1)
Supplement: Supplementary file 2 — Supplementary Figure S1. [file 41598_2022_25195_MOESM2_ESM.docx]

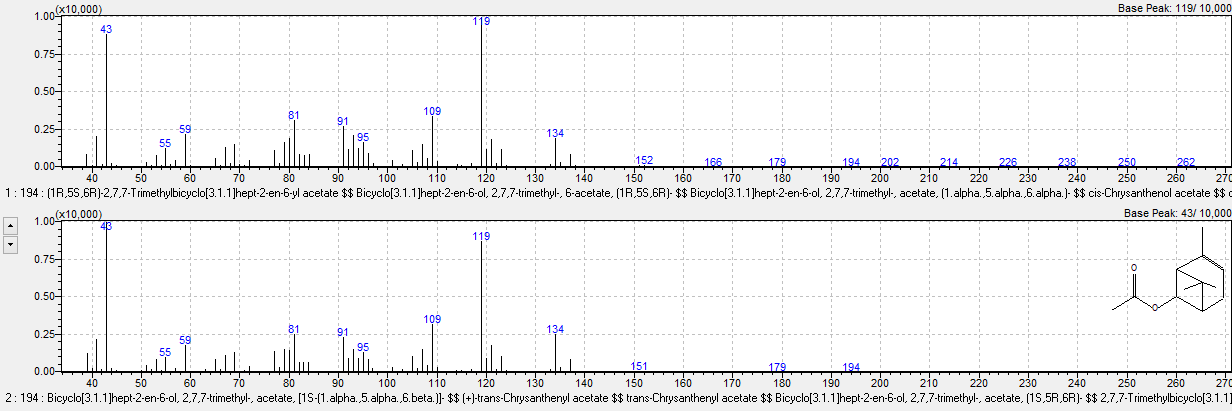


*Cis*-Chrysanthenyl acetate


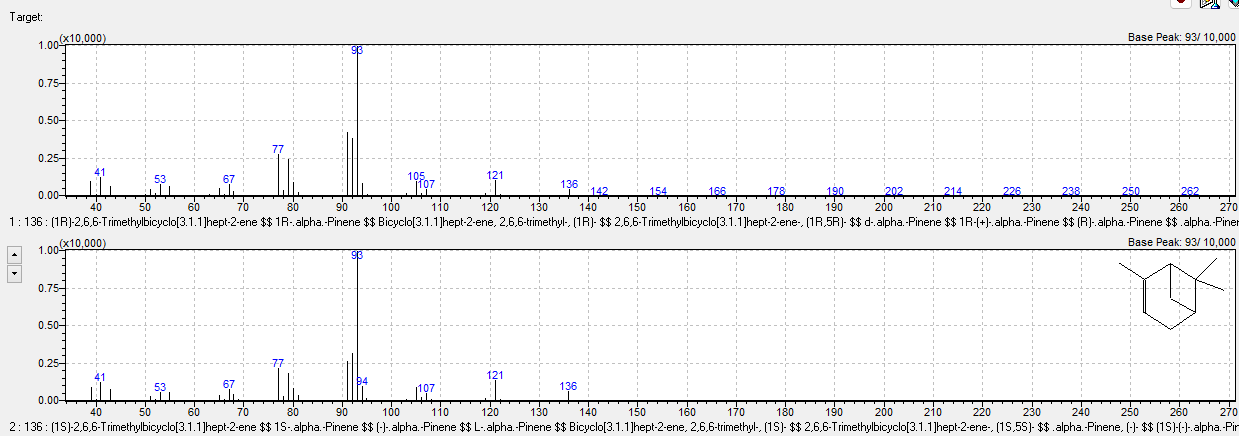


α-Pinene


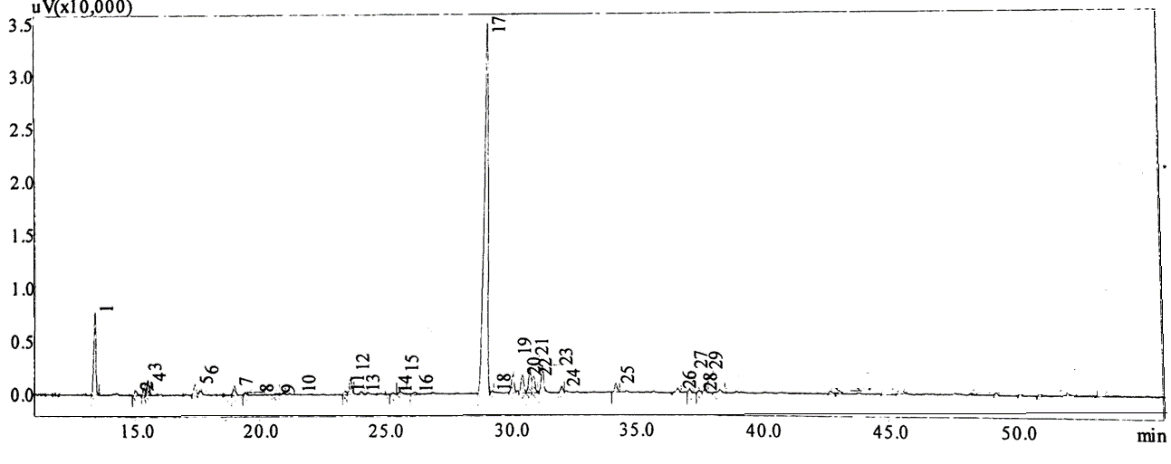


α-pinene

*cis*-chrysanthenyl acetate

**Figure .S1.** GC-mass peaks and mass spectra spectrum of major components of Ducrosia anethifolia seeds EI-MS (70 eV) spectra of main identified compounds are illustrate by database (NIST).
